# Adenovector 26 encoded prefusion conformation stabilized RSV-F protein induces long-lasting Th1-biased immunity in neonatal mice

**DOI:** 10.1038/s41541-020-0200-y

**Published:** 2020-06-12

**Authors:** Leslie van der Fits, Renske Bolder, Marjolein Heemskerk-van der Meer, Joke Drijver, Yolinda van Polanen, Jan Serroyen, Johannes P. M. Langedijk, Hanneke Schuitemaker, Eirikur Saeland, Roland Zahn

**Affiliations:** Janssen Vaccines & Prevention B.V., Leiden, The Netherlands

**Keywords:** Viral infection, Vaccines

## Abstract

While RSV is a major cause of respiratory morbidity in infants, vaccine development is hindered by the immaturity and Th2-bias of the infant immune system and the legacy of enhanced respiratory disease (ERD) after RSV infection following immunization with formalin inactivated (FI)-RSV vaccine in earlier clinical trials. Preclinical studies have demonstrated that an adenoviral vector-based RSV F vaccine candidate (Ad26.RSV.FA2) induces Th1-biased protective immune responses, without signs of ERD upon subsequent RSV challenge. We here developed an Ad26 vector encoding the RSV F protein stabilized in its prefusion conformation (Ad26.RSV.preF). In adult mice, Ad26.RSV.preF induced superior, Th1-biased IgG2a-dominated humoral responses as compared to Ad26.RSV.FA2, while maintaining the strong Th1-biased cellular responses. Similar to adult mice, Ad26.RSV.preF induced robust and durable humoral immunity in neonatal mice, again characterized by IgG2a-dominated RSV F-binding antibodies, and high and stable virus-neutralizing titers. In addition, vaccine-elicited cellular immune responses were durable and characterized by IFN-γ-producing CD4+ and CD8+ T cells, with a profound Th1 bias. In contrast, immunization of neonatal mice with FI-RSV resulted in IgG1 RSV F-binding antibodies associated with a Th2 phenotype, no detectable virus-neutralizing antibodies, and a Th2-biased cellular response. These results are supportive for the clinical development of Ad26.RSV.preF for use in infants.

## Introduction

Respiratory syncytial virus (RSV) is an orthopneumovirus of the *Pneumoviridae* family that causes annual epidemics of acute lower respiratory tract infections, while in addition, reinfection is common^1^. Whereas infections in healthy adults are mild, RSV causes severe disease in immunocompromised persons and elderly^2^. In addition, RSV remains the leading cause of hospitalization due to respiratory disease in infants and children under 5 years of age^3^. Despite the high disease burden of this viral pathogen and a strong incentive for vaccine development, no safe and effective vaccine is yet available^1^. Young infants are an important target population for an infant RSV vaccine, as the disease burden is highest in infants of <6 months of age^4^. Several factors complicate RSV vaccine development for this target population, which include the relative immaturity of the infant immune system and the presence of maternal anti-RSV antibodies that might suppress vaccine-induced immune responses. Another major obstacle in RSV vaccine development is the legacy of vaccine-enhanced respiratory disease (ERD). In clinical trials in the 1960s, children vaccinated with formalin-inactivated (FI-) RSV were not protected against natural infection, but instead experienced more severe illness requiring hospitalizations during subsequent natural infection (including two mortalities), compared to children that did not receive this vaccine^5–8^. In the FI-RSV-vaccinated children, the vaccine induced antibodies with poor virus-neutralizing capacity, and those were associated with immune complex formation and complement deposition in the small airways in the infants who died. Studies in animal models have shown that immunization with FI-RSV induced, next to these poorly neutralizing antibodies, CD4^+^ Th2-biased responses and reduced cytotoxic CD8^+^ T cell priming and evidence of eosinophilia in the lung after RSV challenge^9–16^. The neonatal immune system is characterized by a preferential differentiation of CD4^+^ T helper 2 (Th2) cells, thereby antagonizing T helper 1 (Th1) and cytotoxic responses that are crucial for protection against intracellular pathogens^17^. Thus, the immune profile induced by an ideal RSV candidate for infants should be skewed away from the immune profile induced by FI-RSV and preferably induce Th1 responses, even in the Th2-prone neonatal immune environment.

The RSV fusion surface glycoprotein (RSV F) is an attractive vaccine antigen, since it is the principal target of RSV-neutralizing antibodies^18–20^. This concept is further supported by the clinical efficacy of the currently available anti-F neutralizing monoclonal IgG1 antibody (Palivizumab) for RSV prophylaxis^21,22^. RSV F is present on the viral surface in a metastable prefusion (preF) conformation, which is easily triggered to convert into a postfusion (postF) conformation. RSV preF is highly immunogenic and most RSV-neutralizing antibodies in human sera are directed against the preF conformation. We previously demonstrated that stabilized preF, when given as protein antigen, elicits superior levels of neutralizing antibodies and protection against viral challenge in animal models when compared to non-stabilized F^23^.

Replication-incompetent adenoviral vectors based on serotype 26 have a favorable safety profile and are potent inducers of humoral and cellular Th1 responses in both animals and humans^24–27^. This contrasts to the Th2-biased profile of FI-RSV vaccine which has been associated with ERD upon infection with RSV. Therefore, the expected Th1 immune response elicited by an adenoviral-vectored RSV vaccine likely minimalizes the probability of ERD upon subsequent RSV infection of vaccine recipients. We have previously shown that immunization with Ad26 encoding the wildtype RSV F transgene elicited strong and long-lasting humoral and cellular responses in adult mice and cotton rats^27^. As antibodies directed against preF-specific epitopes are more potent in viral neutralization than those targeting postF^28^ and based on our successful stabilization of the RSV F protein^23^, we have genetically engineered an Ad26 vector encoding the full-length RSV F protein with amino acid substitutions that stabilize the RSV F protein in its prefusion conformation. In our present study we show improved immunogenicity of this Ad26.RSV.preF vector when compared to its parent Ad26.RSV.FA2, and furthermore demonstrate its induction of a potent Th1-biased immune response in neonatal mice.

## Results

### A single immunization with Ad26.RSV.preF induces a Th1-biased immune response in adult mice

**Fig. 1:**
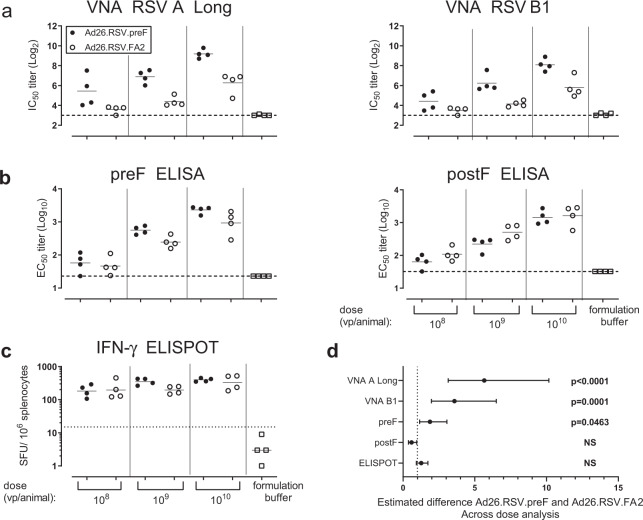
Stabilization of the fusion protein in its prefusion conformation increases humoral responses in adult mice when expressed in an Ad26 vector. Adult BALB/c mice (*n* = 4 per group) were immunized intramuscularly with 1 × 10^8^–1 × 10^10^ vp/animal of Ad26.RSV.preF (•), Ad26.RSV.FA2 (∘) or formulation buffer (▫). Animals were sacrificed 8 weeks post immunization. Serum was analyzed for **a** RSV A Long (left panel) and RSV B1 (right panel) neutralizing antibodies and **b** prefusion (left panel) and postfusion (right panel) IgG antibodies. **c** Splenocytes were stimulated with RSV.F peptides and IFN-γ ELISPOT results are expressed as spot forming units/10^6^ splenocytes. The background levels are indicated with dotted lines and means **a** and **b** or geometric means **c** per group are indicated with horizontal lines. **d** Across dose statistical analysis was performed with Tobit regression and Bonferroni correction. The estimated mean difference between Ad26.RSV.preF and Ad26.RSV.FA2 is indicated with a dot, and the lower and upper confidence limits with whiskers. Statistical differences are indicated. NS: not significant.

Previously we demonstrated that intramuscular immunization of adult mice with Ad26 encoding the wildtype F gene from RSV A2 (Ad26.RSV.FA2) elicited strong humoral and cellular immune responses^27^. We subsequently generated Ad26 with a transgene encoding the RSV A2 F protein but now with mutations that stabilize the protein in its prefusion conformation (Ad26.RSV.preF). Surface expression of prefusion F induced by Ad26.RSV.preF was confirmed by flowcytometric analysis of in vitro-transduced cells using an antibody specific for the prefusion F conformation. Under temperature stress, cells transduced with Ad26.RSV.FA2 loose the preF conformation of the expressed protein, whereas preF expressed on cells transduced with Ad26.RSV.preF appeared more stable. In contrast, total F expression, detected with an antibody recognizing both preF and postF, remained relatively constant for both Ad26.RSV.preF and Ad26.RSV.FA2 (Supplementary Fig. 1). Immunogenicity of Ad26.RSV.FA2 and Ad26.RSV.preF given at 10^8^–10^10^ vp/animal doses was compared in adult mice. Single intramuscular immunization with Ad26.RSV.preF resulted in higher VNA titers against RSV A Long and RSV B1 when compared to Ad26.RSV.FA2 in an across dose statistical comparison. VNA titers were on average 5.6-fold and 3.6-fold higher for Ad26.RSV.preF when compared to Ad26.RSV.FA2, for RSV A Long and RSV B1, respectively (Fig. 1a, d). Levels of IgG-binding antibodies against the preF protein were higher in Ad26.RSV.preF-immunized animals, whereas titers of postF-binding antibodies were similar between Ad26.RSV.preF and Ad26.RSV.FA2 immunized groups (Fig. 1b, d), resulting in a significantly increased ratio between preF-binding and postF-binding antibodies in the former group. Cellular responses were not significantly different in Ad26.RSV.preF and Ad26.RSV.FA2 immunized mice, as determined by ELISPOT (Fig. 1c, d), and by ICS analysis of the CD8+ T cell subsets, whereas CD4+ responses were below background levels for both vectors (Supplementary Fig. 2).

**Fig. 2:**
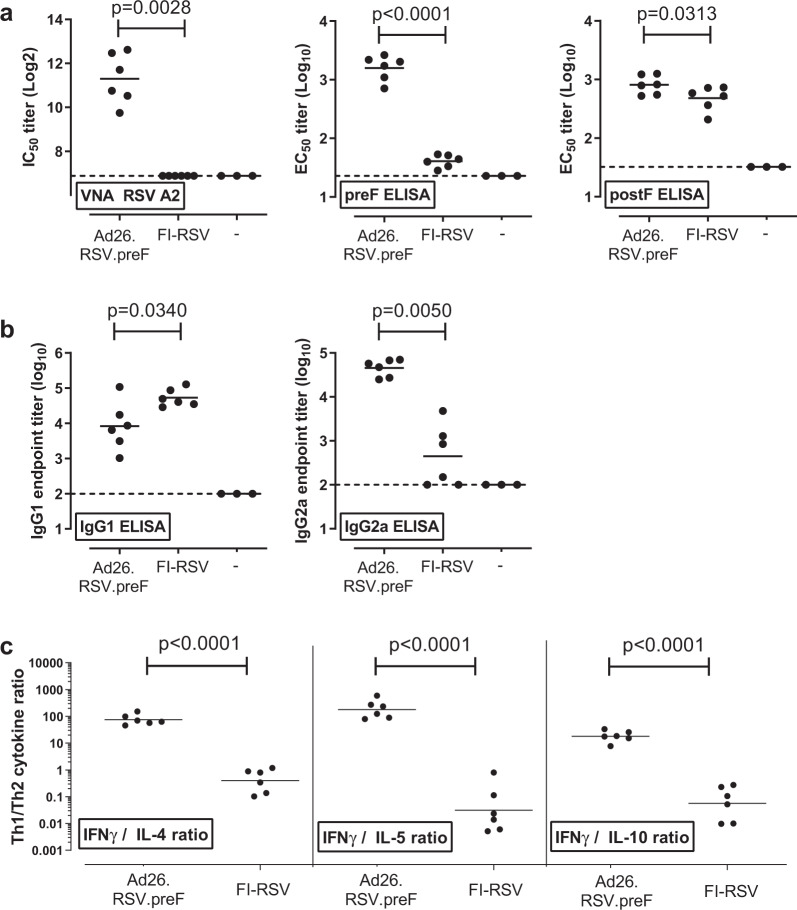
Ad26.RSV.preF polarizes the immune response towards Th1 type in adult mice. Adult BALB/c mice (*n* = 6 per group) were immunized intramuscularly with a single dose of 1 × 10^10^ vp/animal Ad26.RSV.preF, two doses of FI-RSV given 4 weeks apart, or formulation buffer (indicated with -). At 10 weeks post-immunization, animals were sacrificed and sera and spleens were collected. **a** Serum was assayed for VNA against RSV A2 (left panel), or for preF and postF RSV F-binding IgG antibodies (middle and right panel, respectively), **b** RSV-specific IgG1 and IgG2a subclass antibodies against postF as determined by ELISA. **c** Splenocytes were stimulated with RSV F peptide pools. IFN-γ, IL-4, IL-5, and IL-10 secretion in the supernatant was determined by a multiplex ELISA-based analytical method. Ratios between Th1 (IFN-γ) and Th2 (IL-4, IL-5, or IL-10) were calculated for samples with at least one of the values above background levels (95th percentile of unstimulated splenocytes). Means **a** and **b** or geometric means **c** per group are indicated with horizontal lines. Statistical analysis comparing Ad26.RSV.preF and FI-RSV-induced responses was performed using Student’s *t*-test, or Wilcoxon Rank Sum test in case censored values were included (VNA and IgG2a ELISA).

To assess the relative Th1/Th2 skewing, we next compared Ad26.RSV.preF-induced immune responses in adult mice to those induced by FI-RSV given at a dose associated with ERD upon RSV challenge in preclinical animal models^29^. Whereas FI-RSV did not induce VNA titers, VNA titers were induced in all Ad26.RSV.preF-immunized animals. Ad26.RSV.preF-induced antibodies bound both to preF and postF proteins, whereas antibodies induced by FI-RSV were mainly directed against postF (Fig. 2a). Th1/Th2 skewing of the response was assessed by determining the IgG subclass of postF antibodies. Ad26.RSV.preF induced a predominant Th1 response characterized by relatively high levels of IgG2a antibodies. In contrast, the FI-RSV response was characterized by high levels of IgG1 antibodies, and thus a Th2 skewing (Fig. 2b). The difference in IgG2a/IgG1 ratio at 10 weeks post immunization was observed for the complete dose range of Ad26.RSV.preF tested (10^8^–10^10^ vp/animal), and also observed when analyzed 4 weeks post immunization (Supplementary Fig. 3A). In a separate study, the immune response induced by intranasal instillation of live RSV was assessed, as a reference for a response not associated with ERD. Intranasal inoculation of mice with 5 × 10^5^ pfu live RSV A2 resulted in induction of virus-neutralizing antibodies, with a balanced IgG1 to IgG2a response of RSV F-binding antibodies. In addition, systemic IFNγ cellular responses were induced by intranasal application of live RSV A2, although levels were low (Supplementary Fig. 4).

We additionally evaluated cytokine responses in RSV F-stimulated splenocytes from Ad26.RSV.preF and FI-RSV immunized adult mice (Supplementary Fig. 5). Th1/Th2 skewing was determined by calculating the ratio between IFN-γ (as prototypic Th1 cytokine) and IL-4, IL-5, or IL-10 (as prototypic Th2 cytokines). The Th1/Th2 cytokine ratio in samples from mice immunized with FI-RSV was used as reference for a Th2-biased response. Immunization with Ad26.RSV.preF-polarized splenocytes towards IFN-γ production (Supplementary Fig. 5 and Fig. 2c). The difference in cytokine ratio between Ad26.RSV.preF immunization and FI-RSV immunization was highly significant. In addition, this difference was independent of the dose of the Ad26.RSV.preF vector given (ranging from 10^8^ to 10^10^ vp/animal) (Supplementary Fig. 3B).

Due to the superior induction of humoral responses by Ad26.RSV.preF while maintaining the strong Th1-biased cellular responses, this vector was used in subsequent experiments in neonatal mice.

### Immunization of neonatal mice with Ad26.RSV.preF induces long-lasting humoral and cellular immune responses

**Fig. 3:**
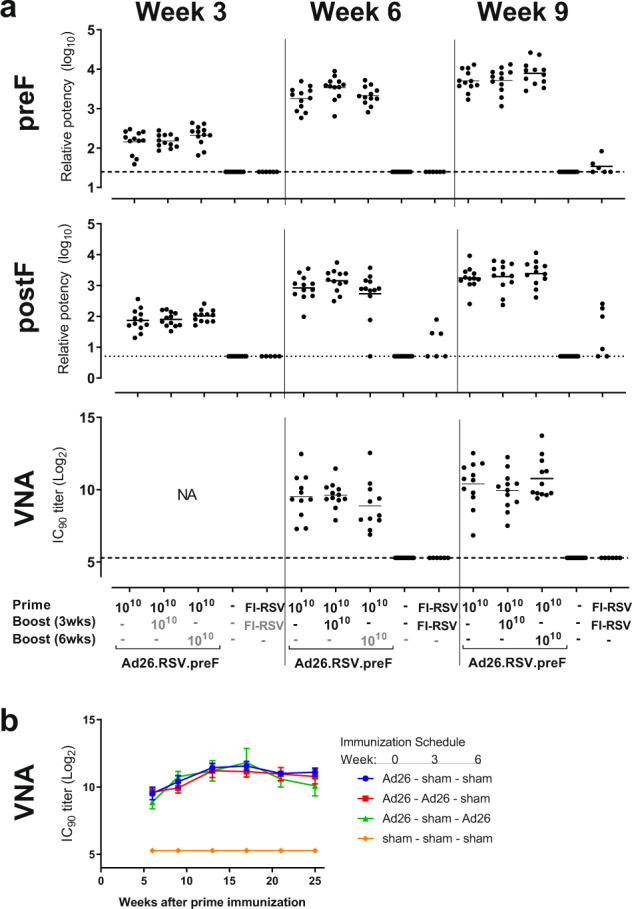
Subcutaneous Ad26.RSV.preF immunization induces durable humoral responses in neonatal mice. Pups were subcutaneously immunized at Day 4–5 after birth, and boosted 3 or 6 weeks later with 10^10^ vp Ad26.RSV.preF in the immunization regimens displayed (*n* = 12 per group). Control groups received no immunization (indicated with - or sham), or FI-RSV (*n* = 6). **a** Prefusion (upper rows) or postfusion (middle rows) RSV F-binding antibody titers were determined by ELISA in serum at 3 weeks (left panels), 6 weeks (middle panels), and 9 weeks (left panels) after prime immunization. RSV CL57 VNA titers (lower row) in sera were determined 6 and 9 weeks after prime immunization. The lower limit of quantification of the assays are indicated with dashed lines, and horizontal bars represent the mean response per group. **b** Mice were followed for 25 weeks post-prime immunization, and RSV CL57 VNA titers were assayed in sera obtained 6, 9 (*n* = 12 per group), 13, 17, 21, and 25 (*n* = 6 per group) weeks after prime immunization. Data are presented as mean ± s.d. per group. ELISA titers are given as the log_10_ value of relative potency, and VNA titers as the log_2_ value of the IC90. NA not analyzed. Statistical analysis was performed with Wilcoxon Rank Sum test and Bonferroni correction, or Tobit regression and Tukey or Tukey–Kramer correction.

As the intended target populations for the Ad26.RSV.preF vaccine candidate include young infants, the immune responses were studied in neonatal mice representing a very immature immune system. Subcutaneous injection of BALB/c neonatal mice with 10^10^ vp Ad26.RSV.preF on Day 4–5 after birth resulted in detectable preF and postF ELISA titers at 3 weeks after immunization, that increased up to 6 and 9 weeks after immunization (Fig. 3a, upper and middle panels, respectively). VNA titers were detected in sera of all Ad26.RSV.preF-immunized neonatal mice at 6 and 9 weeks post immunization, which were significantly higher compared to VNA titers in sera of non-immunized control mice (*p* ≤ 0.0001; Fig. 3a, bottom panel). VNA titers reached plateau levels at 13 weeks post-prime immunization and remained relatively stable for the entire study period (Fig. 3b). The ELISA and VNA titers were not significantly increased in groups that received a boost at 3 or 6 weeks after prime-immunization, when compared to animals that received a single immunization (Fig. 3a, b).

**Fig. 4:**
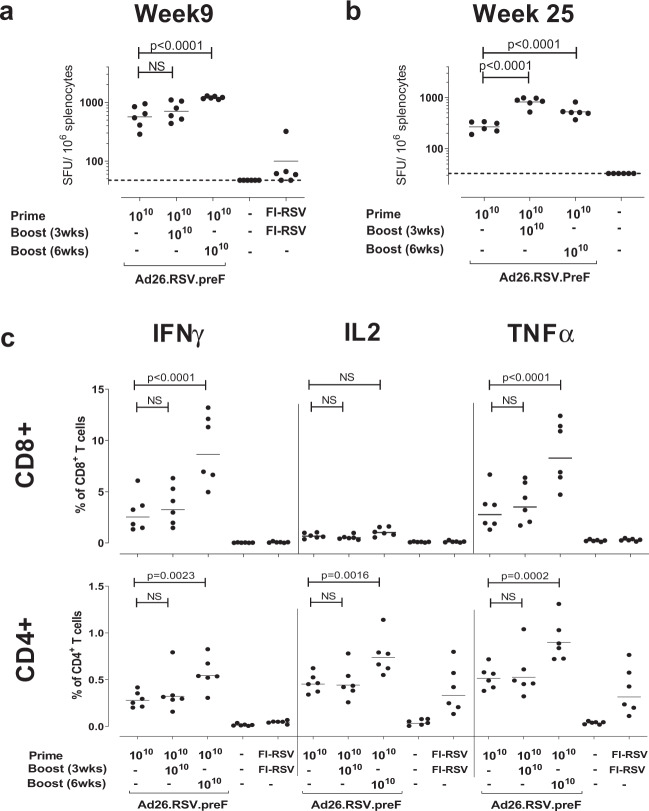
Ad26.RSV.preF induces long lived cellular responses in neonatal mice. Pups were subcutaneously immunized at Day 4 or 5 after birth, and boosted 3 or 6 weeks later with 10^10^ vp Ad26.RSV.preF in the immunization regimens displayed. Control groups received no immunization (indicated with -), or FI-RSV. Splenocytes were isolated 9 weeks (*n* = 6 per group, panels **a** and **c**), or 25 weeks (*n* = 6 per group, panel **b**) after prime immunization, and stimulated overnight with a peptide pool representing the F protein from RSV A2. **a** and **b** IFN-γ ELISPOT results were expressed as spot forming units/10^6^ splenocytes. The background level (95th percentile of unstimulated splenocytes) is indicated with a dotted line. **c** The percentages of CD3^+^CD8^+^ cells (upper panels) or CD3^+^CD4^+^ (lower panels) producing IFN-γ, IL-2, and TNF-α were determined by intracellular cytokine staining. The black bars specify the geometric mean response within each group. Statistical analysis was performed with Wilcoxon Rank Sum test and Bonferroni correction, or Tobit regression and Tukey or Tukey–Kramer correction.

Cellular responses were assessed in splenocytes isolated at 9 weeks after immunization. Stimulation of splenocytes with RSV F peptides resulted in significantly increased numbers of IFN-γ-positive cells in Ad26.RSV.preF-immunized neonatal mice (Fig. 4a), when compared to the non-immunized control group (*p* = 0.0087). A significantly increased response was detected in the group that received a boost at 6 weeks after prime immunization compared to the prime-only immunization group, whereas no significantly increased response was observed in the group that received a boost 3 weeks after prime immunization compared to the prime-only immunization group.

Intracellular cytokine staining (ICS) at week 9 post immunization of RSV F-stimulated splenocytes revealed that significantly increased numbers of IFN-γ, IL-2, and TNF-α-positive cells resided both in the CD8^+^ and CD4^+^ populations after immunization, when compared to the non-immunized control group (*p* = 0.0087 for all comparisons), with higher percentages of IFN-γ and TNF-α-producing cells within the CD8^+^ T cell population when compared to the CD4^+^ T cell population (Fig. 4c). The effect of boost immunizations observed for the ELISPOT responses was confirmed by ICS analysis (Fig. 4c).

At 25 weeks after single Ad26.RSV.preF immunization, high RSV F-specific IFN-γ cellular responses were still detectable and significantly above levels found in animals that received mock immunization (*p* = 0.0087) (Fig. 4b). Prime-boost regimens had a beneficial effect on the durability of the cellular responses with significantly increased numbers of IFN-γ+ splenocytes in the prime-boost groups compared to the prime-only immunization group (Fig. 4b). Again, ICS analysis demonstrated high percentages of IFN-γ^+^, IL-2^+^ and TNF-α^+^ cells mostly within the CD8^+^ T cell population. In addition, percentages of IFN-γ, IL-2, and TNF-α producing CD4^+^ cells were also above baseline levels at 25 weeks after Ad26.RSV.preF immunization. Furthermore, the enhancing effect of a homologous Ad26.RSV.preF boost immunization on cellular responses as observed by ELISPOT analysis, was confirmed by ICS (Supplementary Fig. 6).

### Humoral and cellular immune responses induced by Ad26.RSV.preF in neonatal mice are distinct from those induced by FI-RSV

**Fig. 5:**
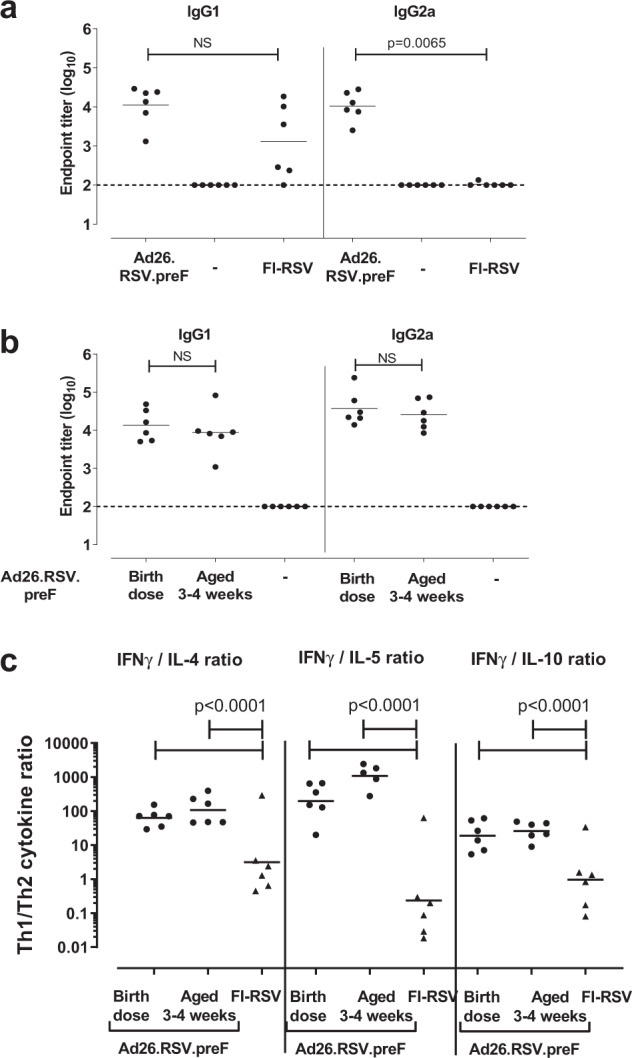
The immune profile induced by Ad26.RSV.preF in neonatal animals deviates significantly from the profile induced by FI-RSV. Neonatal mice (*n* = 6) received a single immunization with 10^10^ vp Ad26.RSV.preF at Day 4 or 5 after birth, or 3 weeks later. Control groups were not immunized (indicated with -), or received a prime-boost immunization with FI-RSV. IgG1 (left panel) and IgG2a (right panel) subclass RSV F-binding antibodies were determined in serum taken 9 weeks **a** or 25 weeks **b** after prime immunization. **c** A subgroup of animals was sacrificed 9 weeks post-prime-immunization and splenocytes were isolated and stimulated with RSV F peptide pools. IFN-γ, IL-4, IL-5, and IL-10 secretion in the supernatant of F-stimulated splenocytes was determined by a multiplex ELISA-based analytical method. Ratios between Th1 (IFN-γ) and Th2 (IL-4, IL-5, or IL-10) were calculated for samples with at least one of the values above background levels (95th percentile of unstimulated splenocytes). Means **a** and **b** and geometric means **c** per group are indicated with horizontal lines. Statistical analysis was performed with Tobit regression and Dunnett or Dunnett–Hsu correction.

Similar to the observations in adult mice (Fig. 2a), a single immunization of neonatal mice with 10^10^ vp Ad26.RSV.preF resulted in high VNA titers with antibodies binding to the F protein in both its prefusion and postfusion conformation, whereas immunization with FI-RSV resulted in no detectable VNA titers, and hardly any prefusion F-binding antibodies (Fig. 3a). PostF-specific binding antibodies induced by Ad26.RSV.preF in neonatal mice were of both IgG1 and IgG2a subclasses. In contrast, FI-RSV induced only RSV F-specific IgG1 antibodies but no detectable IgG2a antibodies, indicative of a Th2-biased profile (Fig. 5a).

This difference in Th1/Th2 skewing by Ad26.RSV.preF and FI-RSV in neonatal mice was further demonstrated by ICS staining of RSV F-stimulated splenocytes. Significantly increased numbers of CD8^+^ T cells producing IFN-γ, IL-2, and TNF-α were observed in Ad26.RSV.preF-immunized mice, when compared to mice immunized with FI-RSV (Fig. 4c). FI-RSV did induce RSV F-specific CD4+ T cells, as evidenced by increased IL-2 and TNF-α production, but without skewing towards the Th1 phenotype as expression of the prototype Th1 cytokine IFN-γ is very limited. In contrast, induction of Th1 skewed CD4+ T cells is observed after immunization with Ad26.RSV.preF, as demonstrated by increased numbers of RSV.F-specific CD4+ T cells positive for IFN-γ, IL2, and TNF-α (Fig. 4c).

Cytokine secretion of RSV F-stimulated splenocytes of immunized neonatal mice was measured by a multiplex ELISA-based method. Whereas high expression of the Th2 cytokines IL-4 and IL-5 was observed in cultures of splenocytes from FI-RSV-immunized neonatal mice, splenocytes from Ad26.RSV.preF-immunized animals secreted increased levels of IFN-γ (Supplementary Fig. 7). Consequently, the Th1/Th2 ratio, as characterized by IFN-γ/IL-4, IFN-γ/IL-5, and IFN-γ/IL-10 ratio, was significantly higher in Ad26.RSV.preF-immunized group compared to FI-RSV-immunized mice (Fig. 5c). Th1/Th2 skewing induced by Ad26.RSV.preF was not altered by boost immunization at 3 or 6 weeks post prime (Supplementary Fig. 8).

To better assess the influence of the age of the mice on the Th1/Th2 profile, we compared immune responses after subcutaneous immunization with Ad26.RSV.preF shortly after birth (on Day 4 or 5), with subcutaneous immunization of mice of 3–4 weeks old in a single study. No meaningful differences were observed in Ad26.RSV.preF-induced cytokine secretion profiles in mice of these age groups, when measured at 9 weeks post immunization (Supplementary Fig. 7), resulting in similar Th1/Th2 cytokine ratios (Fig. 5c). In addition, comparable levels of IgG1 and IgG2a subclass antibodies were observed in both groups when assessed at 25 weeks post immunization (Fig. 5b).

Thus, the Th1-dominated immune response as induced by Ad26.RSV.preF in adult mice is similar to that in neonatal mice immunized with Ad26.RSV.preF at 4–5 days after birth and in young mice immunized at an age of 3–4 weeks.

## Discussion

Although development of an RSV vaccine has been a high priority, there is still no licensed vaccine available. We have previously demonstrated that Ad26 expressing the wildtype F protein from RSV A2 is immunogenic in adult mice and induces both humoral and cellular immune responses^27^. Earlier, we and others showed that a subunit protein vaccine of RSV F stabilized in its prefusion F conformation induces increased quality and quantity of antibodies with RSV-neutralizing activity and an increased protection against RSV challenge in animal models^23,30,31^. These earlier findings are further substantiated by the results described in this manuscript using a membrane-bound prefusion F encoded by Ad26. The observed improved immunogenicity of Ad26.RSV.preF is mainly characterized by increased titers of virus-neutralizing antibodies, linked to increased prefusion F-binding IgG titers, whereas the titers of antibodies binding to postfusion F were not substantially different between Ad26.RSV.preF and Ad26.RSV.FA2 immunized mice. Moreover, similar cellular immunogenicity is observed between Ad26.RSV.preF and Ad26.RSV.FA2, indicating that the processing of the protein and MHC presentation is not altered by the mutations introduced to stabilize F. Vaccine candidates based on non-stabilized F protein have been used in various clinical studies, however clinical efficacy could not be demonstrated in recent large late stage efficacy studies^32^. In contrast, encouraging phase I results have been reported recently for the prefusion F-based subunit vaccine candidate DS-Cav1 that induced high increases in VNA titers in healthy adults^33^.

In the here presented mouse studies, a second dose of Ad26.RSV.preF did not significantly increase the induced humoral responses (Fig. 3). In contrast, in non-human primates we recently observed an increase in (neutralizing) antibody titers after immunization with a second homologous dose of an Ad26-based vaccine candidate^34^. In addition, it has been demonstrated in humans that a homologous two dose immunization regimen with an Ad26 vectorized HIV vaccine candidate did boost the humoral responses^35^. In the current mouse study, the cellular responses were significantly boosted upon a second homologous Ad26.RSV.preF immunization (Fig. 4), suggesting that the lack of a boost effect on the humoral responses is not due to the presence of anti-Ad26 immunity induced by prime immunization (Supplementary Fig. 9).

Immaturity of the newborn immune system might contribute to the increased susceptibility to pathogens and to lower vaccine response when compared to adults. Due to this immaturity, routine childhood vaccination often requires multiple immunization to elicit full immunity. An earlier study with an Ad35 vector-based TB vaccine candidate in young infants (4–6 months of age) indeed demonstrated a lower cellular immune response rate and magnitude when compared to adults^36^. Therefore, potency of novel childhood vaccine candidates should preferentially be tested in neonatal nonclinical models prior to proceeding to clinical testing. However, it should be kept in mind that the ontogeny of the immune system differs between humans and different animal species. Animals with relatively short gestation periods (e.g. mice, rats) have relatively immature immune systems at birth with ongoing immune system development after birth compared to humans^37^. Even in the very immature immune system in mice of 4–5 days of age, Ad26.RSV.preF induced robust and durable immune responses. These responses were both qualitatively and quantitatively similar to those in animals immunized at 3–4 weeks of age when the immune system is considered to be matured^37^ and in adult mice. This suggests that responses to Ad26.RSV.preF are not severely affected by immaturity of the immune system, and that this vector might also be immunogenic in young infants. Recently, the potency of various RSV vaccine candidates was studied in larger infant animal models, for which the immune system ontogeny might better parallel the human situation. However, these studies do not describe a direct comparison of the vaccine potency or Th1/Th2 bias of the response between infants and adults of the same species. ΔF/TriAdj, a truncated adjuvanted fusion protein, was shown to be immunogenic in lambs, both in the presence and absence of maternal anti-RSV antibodies^38^. Efficacy of adjuvanted bovine RSV F-based vaccine candidates has been demonstrated in calves in a species homologous bovine RSV infection model^39,40^. Interestingly, human RSV F, N and M2-1 antigens expressed by a chimpanzee Adenoviral vector were immunogenic and protected calves against challenge with the heterologous bovine RSV^41^.

ERD is a serious concern in neonates and young infants. ERD is, among other things, characterized by a Th2-biased immune response. Neonates, both mouse and human, are prone to mount Th2-biased responses, which for mice was shown to be due to epigenetic imprinting in CD4+ T cells^42^. However, neonates are able to mount a Th1-biased response when given the right stimuli^43^. As Th2-biased responses are undesirable for an RSV vaccine intended for the infant population, we carefully examined the Th1/Th2 skewing of the response in neonatal mice immunized with Ad26.RSV.preF. In mice, a Th1-biased immune response is dominated by antibodies of the IgG2a subclass, whereas IgG1 antibodies are a hallmark of a Th2-biased response making the IgG2a/IgG1 antibody ratio an indication for Th1/Th2 skewing^44^. Upon FI-RSV immunization, a relatively low IgG2a/IgG1 antibody ratio was observed in both adult and neonatal mice, indicative for a Th2-biased response, although the absolute IgG titers differed considerably between both age groups (Figs. 2b and 5a). Antibodies induced by Ad26.RSV.preF were strongly dominated by IgG2a in adult mice (Fig. 2b), whereas a more balanced response between IgG2a and IgG1 was observed in neonatal mice (Fig. 5a). Thus, although differences were observed in the Ad26.RSV.preF response between adult and neonatal mice, the subtyping of the antibody response strongly deviates away from the Th2-biased profile induced by FI-RSV for both age groups. Cytokine profiles induced by FI-RSV were markedly similar between adult and neonatal mice, with relatively high levels of IL-2, IL-5, IL-6, and IL-10 (Supplementary Figs. 5 and 7). Completely different profiles were induced by Ad26.RSV.preF, again very similar in adult and neonatal mice, with high levels of IFN-γ and TNF-α, and background levels for IL-5 and IL-10 (Supplementary Figs. 5 and 7). Thus, our results demonstrate a profound Th1 bias of the immune response in both adult and neonatal mice after Ad26.RSV.preF immunization. These data are in line with previously published data on newborn rhesus monkeys showing that an Ad26 vectored vaccine induces CD4+ and CD8+ T cells producing IFN-γ when given on the day of birth^45^. Altogether this indicates that the Adenoviral vector platform is able to overcome the Th2 bias of the neonatal immune system, and instead induces Th1-biased responses. Another feature of the FI-RSV-induced immune response thought to contribute to the predisposition to ERD is the induction of antibodies with poor neutralizing capacity. We previously demonstrated that Ad26 encoding wildtype RSV F did not induce any signs of ERD in an adult cotton rat RSV challenge model^27^. We here demonstrate that Ad26.RSV.preF induces even higher levels of neutralizing antibodies accompanied by a higher ratio of RSV neutralizing to RSV postF-binding antibodies, combined with a similar Th1-biased cellular response when compared to Ad26 encoding wildtype RSV F. As these characteristics skew the response away from FI-RSV-induced immune responses, this is highly suggestive of a lack of ERD predisposition by Ad26.RSV.preF.

In recent clinical studies, the safety and induction of durable anti-RSV F cellular and humoral responses were demonstrated in healthy adults and elderly immunized with Ad26 encoding RSV F (11th International RSV Symposium Asheville, 2018). In addition, various animal and human studies have demonstrated the safety and clinical immunogenicity of adenoviral vector vaccines in infants and young children^36,46,47^. Combined with our results demonstrating that a single immunization with Ad26.RSV.preF in neonatal animals induces robust and durable neutralizing antibodies and cellular Th1-biased immune responses, indicative for a very low likelihood of ERD predisposition, further clinical development of this vector for prevention of RSV in young infants was initiated (NCT03606512, Clinical Trials.gov). In addition, use of this vector for other target populations is currently clinically explored (ClincialTrials.gov, NCT03982199).

## Methods

### Ethics statement

All animal studies were conducted in compliance with all relevant ethical regulations according to the Dutch Animal Experimentation Act and the Guidelines on the Protection of Experimental Analysis by the Council of the European Committee after approval by the Centrale Commissie Dierproeven and the Dier Experimenten Commissie.

### Animals and immunizations

For studies with adult mice, 6–8-weeks-old-specific pathogen-free (SPF) female BALB/c mice purchased from Charles River were given a single intramuscular (i.m.) immunization (50 μl/leg) with 1 × 10^8^–1 × 10^10^ virus particles (vp) of replication-incompetent recombinant Ad26 vectors encoding RSV preF (Ad26.RSV.preF), wildtype RSV F (Ad26.RSV.FA2) or formulation buffer. A control group was immunized with FI-RSV (at a dose known to elicit ERD in vivo^29^, kind gift of K. Yim, Sigmovir Biosystems) in a prime-boost regimen with 4 weeks interval. Immune responses were evaluated at 4, 8, or 10 weeks post immunization. In a separate experiment, adult SPF female BALB/c mice (Charles River) were intranasally exposed to 5 × 10^5^ pfu RSV A2 or culture medium. Humoral responses were analyzed at 4, 8, and 10 weeks after exposure, and cellular responses were assayed on splenocytes isolated at sacrifice 10 weeks after exposure.

For studies with neonatal mice, pregnant SPF BALB/c mice (Charles River) were purchased and animals were randomized into groups by gender and litter. On Day 4–5 after birth, neonatal mice were prime-immunized with 1 × 10^10^ vp of Ad26.RSV.preF subcutaneously (s.c.) in the scuff of the neck (50 µl), and boosted 3 or 6 weeks later where indicated. Control groups received no immunization, or prime-boost immunization with FI-RSV. Cellular responses were evaluated at 9 and 25 weeks and humoral responses at 3, 6, 9, 13, 17, 21, and 25 weeks after prime immunization.

Serum was obtained by submandibular bleeding or heart puncture under isoflurane anesthesia. Spleens were removed aseptically during necropsy after cervical dislocation.

### Adenoviral vectors

Replication-incompetent, E1/E3-deleted recombinant adenoviral vectors based on Ad26 were engineered using the AdVac® system^48^. Ad26.RSV.FA2, encoding a codon optimized full length RSV F gene from the RSV-A2 strain, is described by Widjojoatmodjo et al.^27^ An optimized Ad26 vector was constructed containing the full length codon optimized F gene from the RSV-A2 strain stabilized in its prefusion conformation using amino acid substitutions^23^.

### In vitro characterization of membrane expressed fusion protein

Cultured A549 cells were transduced with Ad26.RSV.FA2, Ad26.RSV.preF, or empty Ad26 vector at a multiplicity of infection (MOI) of 5000 or 20,000. Forty-eight hours after Adeno infection, cells were harvested and subjected to temperature shock by incubation for 10 min at the temperatures indicated. Temperature-shocked cells were stained using Alexa647-conjugated CR9501 or CR9503 antibodies, detecting prefusion F or both prefusion and postfusion F, respectively. Dead cells were stained using propidium iodide (PI). Cells were acquired on a FACSCanto, and results were analyzed using FlowJo software. Live cells were gated based on forward and sideward scatter, and PI negativity. The percentage of cells positive for Alexa647 signal was calculated relative to the percentage of positive cells at 37 °C.

### RSV prefusion and postfusion F ELISA

IgG antibodies to RSV prefusion and postfusion F were measured by enzyme-linked immunosorbent assay (ELISA). Plates were coated with anti-RSV F (Synagis) followed by preF^23^ or postF protein, and serially diluted serum. Mouse RSV F-specific antibodies were detected by horse radish peroxidase (HRP)-labeled anti-mouse IgG and the wells were developed with O-phenylenediamine dihydrochloride (OPD) substrate. The reaction was stopped by adding 1 M H_2_SO_4_, the optical density was measured at 492 nm, and titers expressed as log10 of the half-maximal effective concentration (EC50).

Alternatively, RSV preF and postF mouse IgG antibodies were detected by ELISA using streptavidin-coated plates incubated with biotin-labeled preF^23^ or postF protein, followed by serially diluted serum. Mouse RSV F-specific antibodies were detected by HRP-labeled anti-mouse IgG and the wells were developed with LumiGlo. The luminescence signal was measured and relative potency against a standard sample was calculated.

### Serum RSV F-specific IgG subclass ELISA

RSV F-specific IgG1 and IgG2a antibody titers in mouse serum were measured using ELISA. Serial dilutions of serum samples were added to plates coated with RSV A2 postfusion F protein. RSV-specific antibody subclasses were quantified using HRP-labeled anti-mouse IgG1or IgG2a. Endpoint titers were calculated by linear interpolation.

### Virus neutralization assay (VNA)

VNA titers against RSV Long or RSV B1 were determined by a microneutralization assay. Heat-inactivated serial diluted sera were mixed with RSV Long or B1 and incubated for one hour at 37 °C. Subsequently, VERO cells were added. Three days later monolayers were washed and fixed, and RSV replication was determined by measuring RSV F protein expression using a biotin-conjugated anti-F monoclonal antibody. Streptavidin–HRP was added to the wells, and the wells were developed with OPD substrate. VNA titers were calculated as the antibody concentration that caused a 50% reduction in the OD450, expressed as IC50 titers (log2).

Alternatively, VNA against RSV A2 or clinical isolate CL57 was determined using recombinant RSV viruses. These viruses encode for the firefly luciferase (FFL), harbor the G and F genes of RSV strains A2 or CL57 in an RSV A2 backbone, and have been derived via a BAC-based reverse genetics system as described in Hotard et al.^49^. Serial dilutions of heat-inactivated sera were mixed with RSV A2 or CL57 FFL and incubated prior to the addition of A549 cells. After an incubation period of 20 h, neolite substrate was added and the luminescence signal was measured. VNA titers were calculated as the antibody concentration that caused a 90% reduction in luminescence (expressed as IC90 values (log2)).

### Enzyme-linked immunospot assay (ELISpot)

An ELISpot assay was used to determine the number of F protein-specific IFN-γ-secreting T cells in the spleen. Splenocytes were stimulated overnight with a peptide pool consisting of 15-mer peptides with an 11-amino-acid-overlap, spanning the whole sequence of the F protein of RSV A2. The numbers of spot-forming units (SFU) were determined using mouse IFN-γ ELISpot kit (MabTech), according to the manufacturer’s instruction, and calculated to numbers of SFU per 10^6^ cells. Background levels were calculated as the 95% percentile of the SFU observed in non-stimulated splenocytes.

### Intracellular cytokine staining

Antigen-specific cellular immune responses were measured by ICS. Splenocytes were stimulated overnight with an RSV F peptide pool, hamster-anti-mouse CD28 and rat-anti-mouse CD49d. GolgiPlug was added after 1 h. Samples were stained by Amine Reactive Violet dye (Invitrogen) for dead cell discrimination. Anti-mouse CD16/CD32 antibodies were used to block Fc receptors and cells were stained with anti-CD3-FITC, anti-CD4-PerCpCy5.5, and anti-CD8-APC-H7. The cells were then permeabilized with Cytofix/Cytoperm and subsequently intracellularly stained using anti-IFN-γ-PE, anti-TNF-α-PE-Cy7, and anti-interleukin (IL)-2-APC antibodies. The percentage of CD3^+^CD4^+^ and CD3^+^CD8^+^ T cells expressing IFN-γ, TNF-α, or IL-2 was quantified by flow cytometry using BD FACS Canto II. All reagents were from BD Biosciences. Analysis of flow cytometric data was performed in FlowJo software version 9.6.1 (Ashland, OR). An example of the gating strategy applied is shown in Supplementary Fig. 10.

### Th1/Th2 cytokine analysis

To analyze the Th1/Th2 skewing after immunization, cytokine secretion was assessed in culture supernatant of stimulated splenocytes. Single cell suspensions of splenocytes were stimulated for 48 h with a peptide pool representing the complete F protein in RPMI medium without fetal bovine serum. The culture supernatant was harvested and analyzed for the presence of Th1 and Th2 cytokines (IFN-γ, TNF-α, IL1-β, IL-2, IL-4, IL-5, IL-6, IL-10, IL-12p70, KC/GRO) using the V-PLEX Proinflammatory Panel 1 Mouse kit (MSD), according to the manufacturer’s instructions.

### Statistical analysis

For the across dose comparisons, an analysis-of-variance (ANOVA) for potentially censored measurements (Tobit regression) with a Bonferroni correction was implemented.

For other analyses, a Wilcoxon Rank Sum test with a Bonferroni correction, was used in case of completely censored groups, or a Student’s *T* test or Tobit regression model was used with Tukey, Tukey–Kramer, Dunnett or Dunnett–Hsu corrections.

ELISPOT SFU and Th1/Th2 cytokine ratios were log10 transformed, and ICS cell counts were square root transformed for statistical analysis.

All comparisons were performed using two-sided tests, at the 5% overall significance level.

## Material availability

Biological materials described are proprietary material of Janssen, and can therefore not be made available to external parties.

### Reporting summary

Further information on research design is available in the Nature Research Reporting Summary linked to this article.

## Supplementary information

Supplementary Information

Reporting Summary

## Data Availability

All data to understand and assess the conclusions of this research are available in the main text and Supplementary Information. The raw data that support the findings of this study are available from the corresponding author upon reasonable request.
