# Supplementary material for: Adenovector 26 encoded prefusion conformation stabilized RSV-F protein induces long-lasting Th1-biased immunity in neonatal mice
Source: NPJ Vaccines. 2020 Jun 12;5:49. doi: 10.1038/s41541-020-0200-y (PMC7293210; doi:10.1038/s41541-020-0200-y)
Supplement: Supplementary file 1 — Supplementary Information [file 41541_2020_200_MOESM1_ESM.pdf]

SUPPLEMENTARY INFORMATION

For manuscript "Adenovector 26 encoded prefusion conformation stabilized RSV-F protein induces long-lasting Th1-biased immunity in neonatal mice" by Leslie van der Fits et al., NPJ Vaccines, 2020.

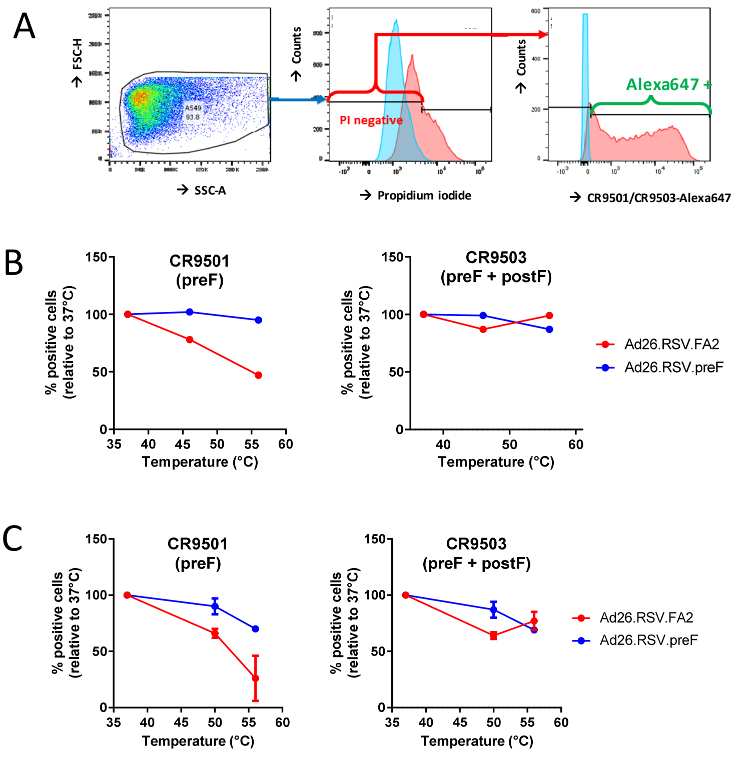

Supplementary Figure 1: Ad26.RSV.preF induces expression of stable preF on the surface of transduced cells. A549 cells were transduced by Adenoviral vectors harboring wildtype or preF. After 48 h, cells were detached, temperature stressed for 10 minutes, and stained with a viability dye (propidium iodide, PI), Alexa647-labeled CR9501 (detecting preF) or CR9503 (detecting preF and postF) monoclonal antibodies. (A) Exemplified gating strategy demonstrating identification of A549 cells based on forward and side scatter characteristics. Live cells were gated based in negativity for staining with PI. CR9501 or CD9503 positivity was determined on live cells, with gates based on the signal of unstained cells, indicated with blue histograms. Percentage of the gated viable cells staining positive for CR9501 or CR9503 were expressed relative to the percentage positive cells at 37°C, and presented in panels B and C. (B) Cells were transduced with Ad26 vectors at a multiplicity of infection (MOI) of 20.000, and stressed at temperatures of 37, 46 and 56 °C. (C) Cells were transduced in duplicate with Ad26 vectors at a multiplicity of infection (MOI) of 5.000, and stressed at temperatures of 37, 50 and 56 °C. Means +/- standard deviations are indicated.

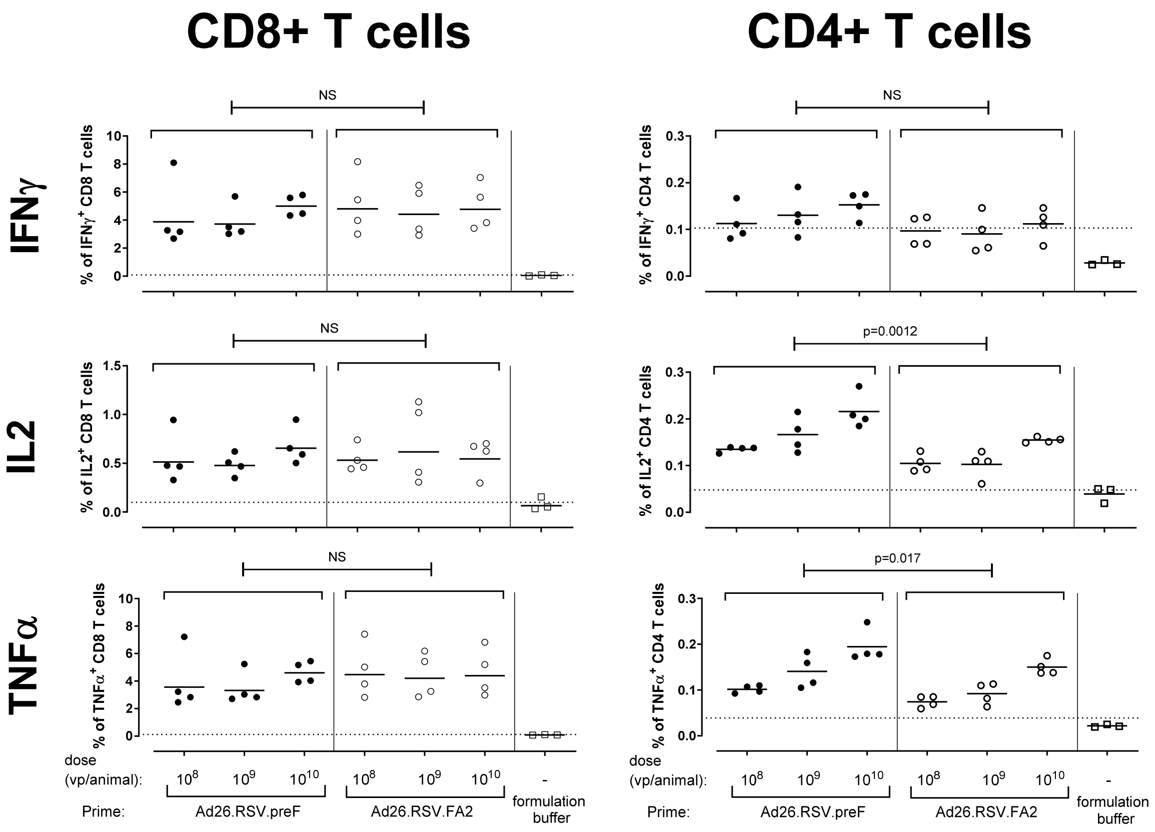

Supplementary Figure 2: Ad26.RSV.preF and Ad26.RSV.FA2 induce similar CD8 T cells responses, whereas CD4 T cell responses are low in adult mice. Adult BALB/c mice (n=4 per group) were immunized intramuscularly with $1x10^8$ to $1x10^{10}$ vp/animal of Ad26.RSV.preF (closed circles), Ad26.RSV.FA2 (open circle) or formulation buffer (open squares). Animals were sacrificed 8 weeks post immunization. Splenocytes were stimulated with RSV.F peptides and the percentages of $CD3^+CD8^+$ cells (left panels) or CD3+CD4+ (right panels) producing IFN-γ (upper panel), IL-2 (middle panel), and TNF-α (lower panel) were determined by intracellular cytokine staining. The black bars specify the geometric mean response within each group. Background staining (mean + 3x standard deviation of unstimulated splenocytes) is indicated with a horizontal dotted line. Note: analysis of 1 sample of the formulation buffer control group failed. Across dose statistical analysis was performed with Tobit regression and Bonferroni correction.

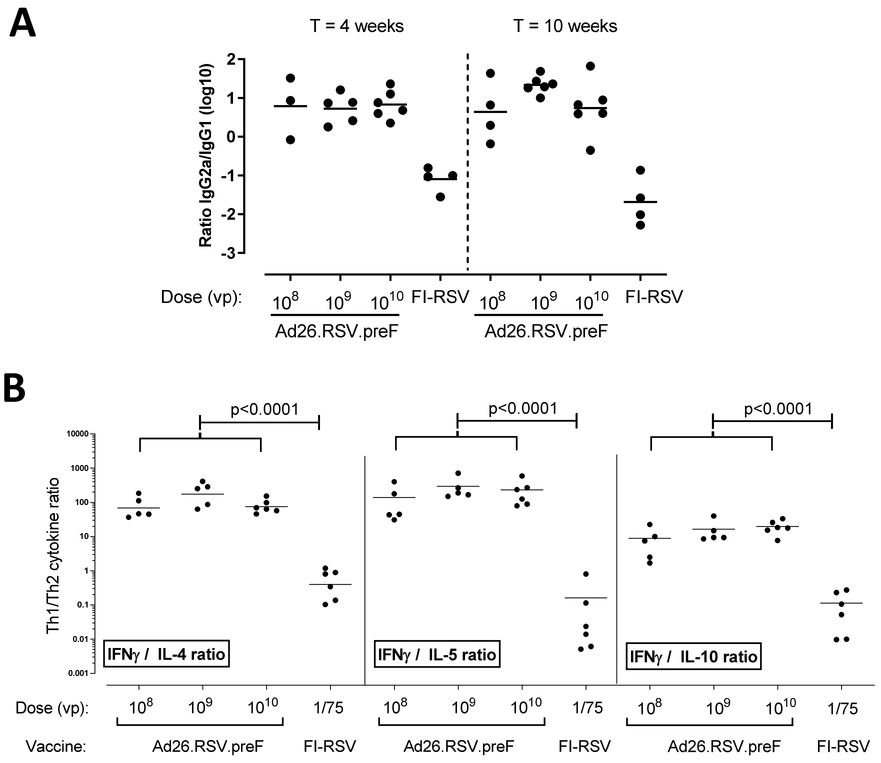

Supplementary Figure 3: Th1-biased immune responses is not affected by the Ad26.RSV.preF dose. Adult BALB/c mice (n=6 per group) were immunized intramuscularly with a single dose of $1x10^8$, $1x10^9$ or $1x10^{10}$ vp/animal Ad26.RSV.preF or with 2 doses of FI-RSV with a 4 week interval. At 4 weeks post prime immunization plasma was collected, and 10 weeks post-immunization, animals were sacrificed and serum and spleens were collected. (A) Plasma (4 weeks) or serum (10 weeks) was assayed RSV-specific IgG1 and IgG2a subclass antibodies against postF, and IgG2a/IgG1 ratios were calculated. (B) Splenocytes were stimulated with RSV.F peptides. IFN-γ, IL-4, IL-5 and IL-10 secretion in the supernatant was determined by a multiplex ELISA-based analytical method. Ratios between Th1 (IFN-γ) and Th2 (IL-4, IL-5 or IL-10) were calculated for samples with at least one of the values above background levels (95 percentile of unstimulated splenocytes). Means (panel A) or geometric means (panel B) per group are indicated with horizontal lines. Statistical analysis comparing Ad26.RSV.preF and FI-RSV induced responses was performed using Student's t-test.

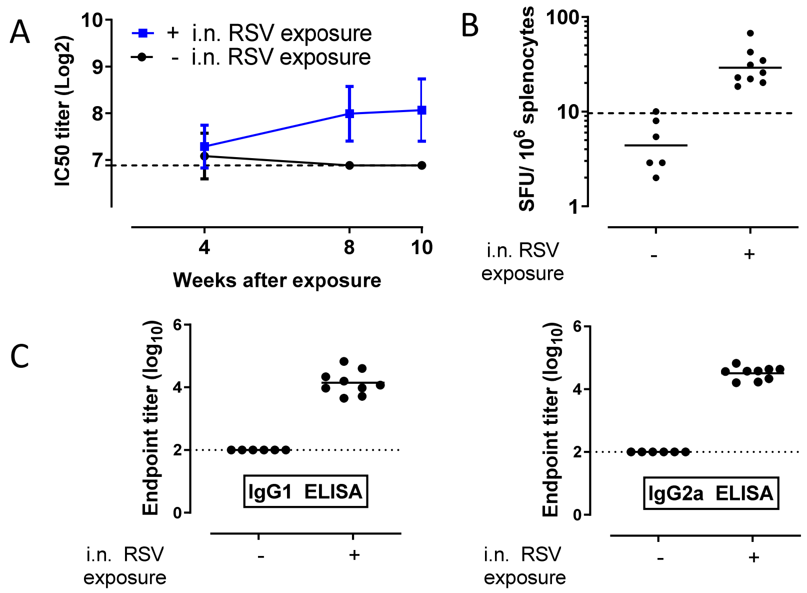

Supplementary Figure 4: Characterization of immune responses induced by intranasal instillation of live RSV A2. Adult BALB/c mice were intranasally (i.n.) exposed to $5x10^5$ pfu RSV A2 (n=9), or culture medium (n=6). At different time points after exposure, mice were bled and serum was collected. At 10 weeks post-exposure, animals were sacrificed and serum and spleens were collected. (A) Serum isolated was assayed for VNA against RSV A2. Means +/- SD per group are indicated. (B) Splenocytes were stimulated with RSV.F peptides and IFN-γ ELISPOT results are expressed as spot forming units/10^6 splenocytes. (C) RSV-specific IgG1 and IgG2a subclass antibodies against postF were determined by ELISA in serum isolated 10 weeks after exposure. The background levels are indicated with dotted lines, and means (A and C) or geometric means (B) per group are indicated with horizontal lines.

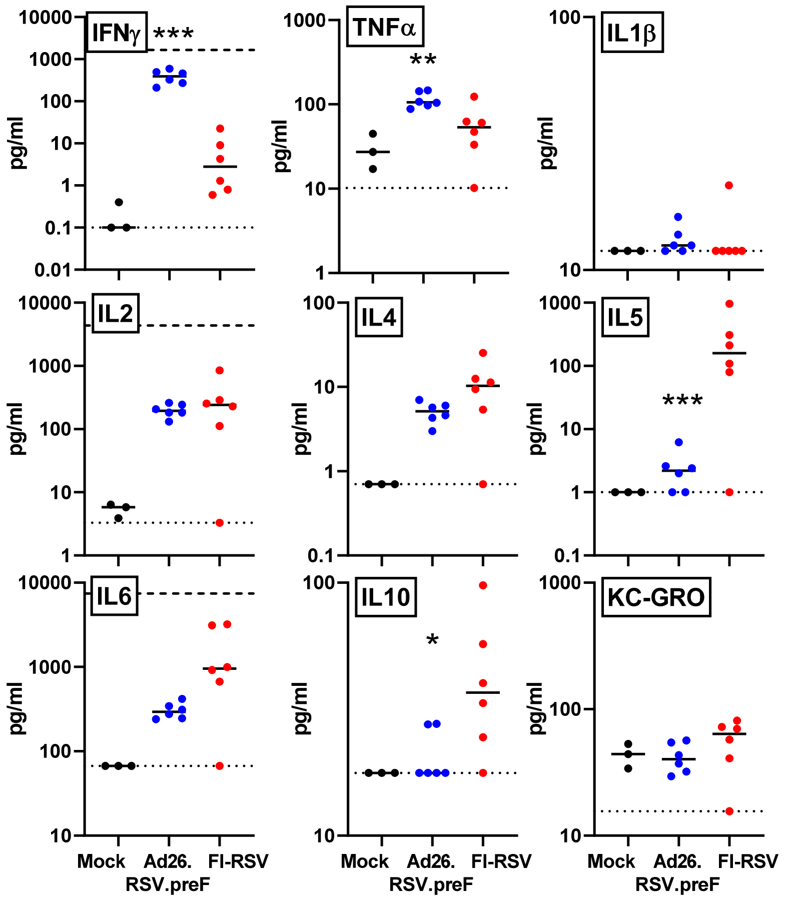

Supplementary Figure 5: Cytokine secretion in RSV F stimulated splenoctyes from immunized adult mice. Adult BALB/c mice were immunized intramuscularly with $1x10^{10}$ vp/animal of Ad26.RSV.preF (n=6 per group, indicated in blue), FI-RSV (n=6, indicated in red) or with formulation buffer (mock, n=3, indicated in black). At 10 weeks post-immunization, animals were sacrificed and splenocytes were stimulated with RSV F peptides. Cytokine secretion into the supernatant was determined by a multiplex ELISA-based analytical method in pg/ml. Mean secretion per group is indicated with an horizontal line. IL12p70 secretion was also measured, but found hardly above the Lower Limit of Quantification (LLoQ) of the assay, and therefore not shown. Background is defined as the 95% percentile secretion of unstimulated splenocytes, indicated with a dotted line. Upper Limit of Quantification (ULoQ) was defined as one dilution step above the highest sample of the standard series, indicated with a dashed line, where applicable. Samples at or below background were set on background levels. Cytokine secretion in Ad26.RSV.preF immunized animals was compared to secretion in FI-RSV immunized animals (Tobit regression). Statistically significant differences between Ad26.RSV.preF and FI-RSV immunized animals are indicated (* $p \leq 0.05$; ** $p \leq 0.01$; *** $p \leq 0.001$).

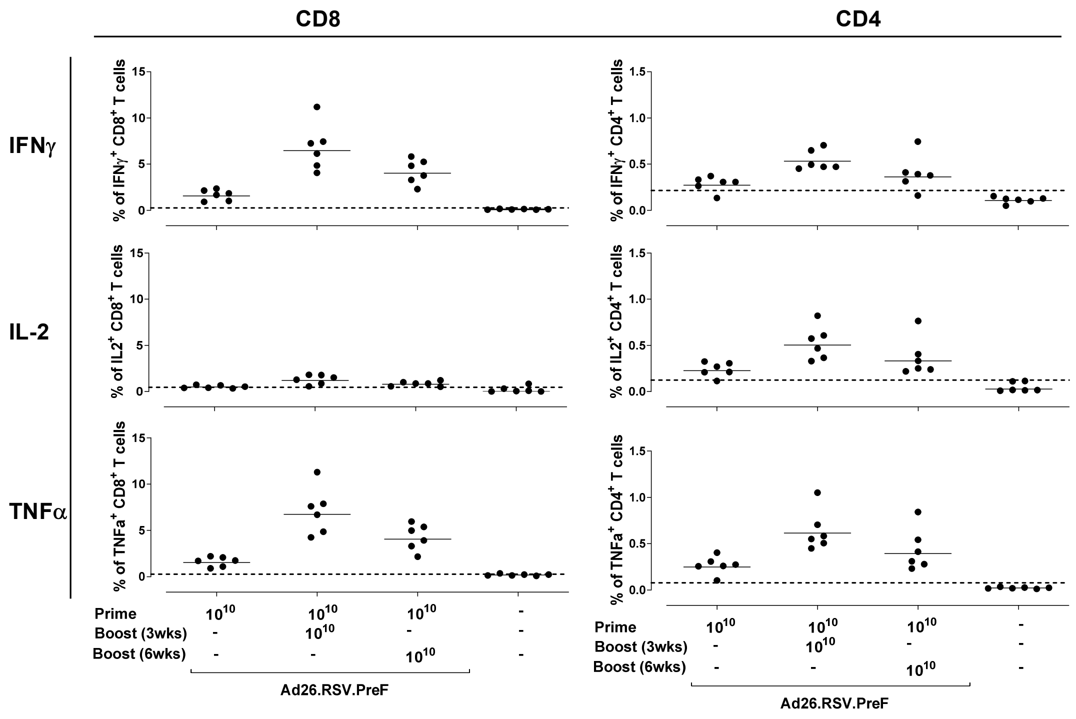

Supplementary Figure 6: Ad26.RSV.preF-induced durable T cell responses in neonatal mice. Pups were subcutaneously immunized at Day 4 or 5 after birth (indicated with prime), and boosted 3 or 6 weeks later with 10^{10} vp Ad26.RSV.preF in the immunization regimens displayed. Control groups received no immunization (indicated with -). Splenocytes were isolated 25 weeks (n=6 per group) after immunization, and stimulated overnight with a peptide pool representing the F protein from RSV A2. The percentages of $CD3^+CD8^+$ cells (left panels) or $CD3^+CD4^+$ (right panels) producing IFN-γ, IL-2, and TNF-α were determined by intracellular cytokine staining. The black bars specify the geometric mean response within each group. Background staining (mean + 3x standard deviation of unstimulated splenocytes) is indicated with a horizontal dotted line.

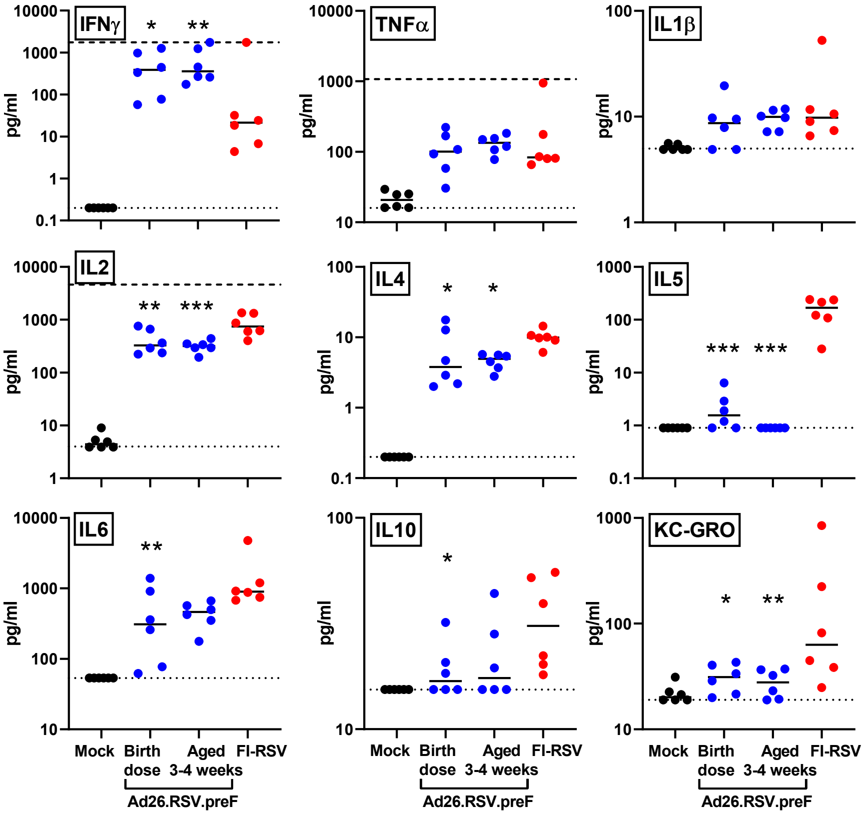

Supplementary Figure 7: Cytokine secretion in RSV F stimulated splenoctyes from immunized neonatal mice. Neonatal BALB/c mice were immunized subcutaneously with $1x10^{10}$ vp/animal of Ad26.RSV.preF at Day 4 or 5 after birth or 3 weeks later (indicated in blue). Control groups were immunized with FI-RSV (indicated in red) or were not immunized (indicated with Mock, in black) (n=6 per group). At 9 weeks post-immunization, animals were sacrificed and splenocytes were stimulated with RSV F peptides. Cytokine secretion into the supernatant was determined by a multiplex ELISA-based analytical method (pg/ml). Mean secretion per group is indicated with an horizontal line. IL12p70 secretion was also measured. but found hardly above the Lower Limit of Quantification (LLoQ) of the assay and therefore not shown. Background is defined as the 95% percentile secretion of unstimulated splenocytes, indicated with a dotted line. Upper Limit of Quantification (ULoQ) was defined as one dilution step above the highest sample of the standard series, indicated with a dashed line, where applicable. Samples at or below background were set on background levels, whereas samples above ULoQ were set at ULoQ. Cytokine secretion in Ad26.RSV.preF immunized animals was compared to secretion in FI-RSV immunized animals (Tobit regression and Dunnett or Dunnett-Hsu correction). Statistically significant differences are indicated (* $p \leq 0.05$; ** $p \leq 0.01$; *** $p \leq 0.001$).

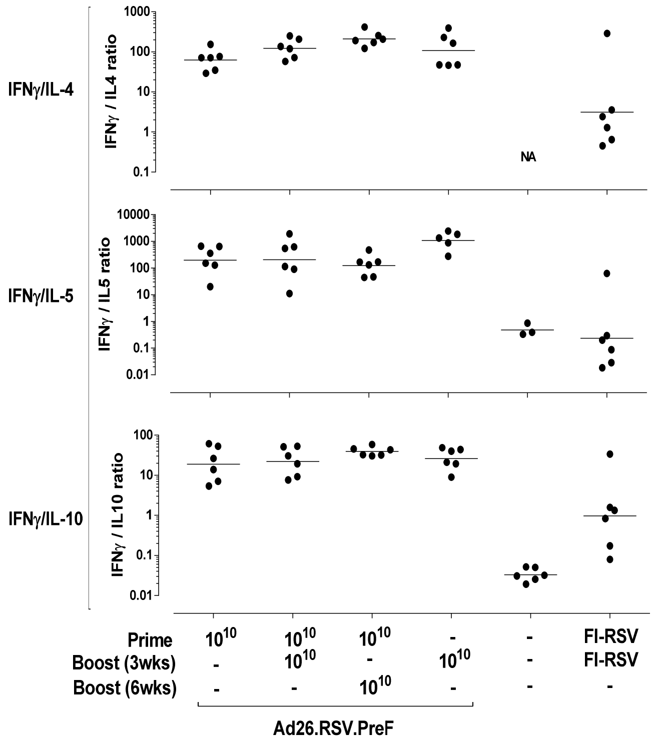

Supplementary Figure 8: Ad26.RSV.preF induces a Th1 cytokine profile in neonatal mice, irrespective of the immunization regimen. Pups (n=6) received a prime immunization with 10^{10} vp Ad26.RSV.preF at day 4 or 5 after birth (indicated with 'prime'), and optional boost immunizations 3 or 6 weeks later, as indicated at the bottom of the figure. '–' indicates that no immunization was given. Control groups were not immunized, or received a prime-boost immunization with FI-RSV. Animals were sacrificed 9 weeks post-prime-immunization and splenocytes were isolated and stimulated with RSV F peptides. IFN-γ. IL-4. IL-5 and IL-10 secretion in the supernatant of F stimulated splenocytes was determined by a multiplex ELISA-based analytical method. Ratios between Th1 (IFN-γ) and Th2 (IL-4, IL-5 or IL-10) were calculated for samples with at least one of the values above background levels (95 percentile of unstimulated splenocytes). Geometric means per group are indicated with horizontal lines.

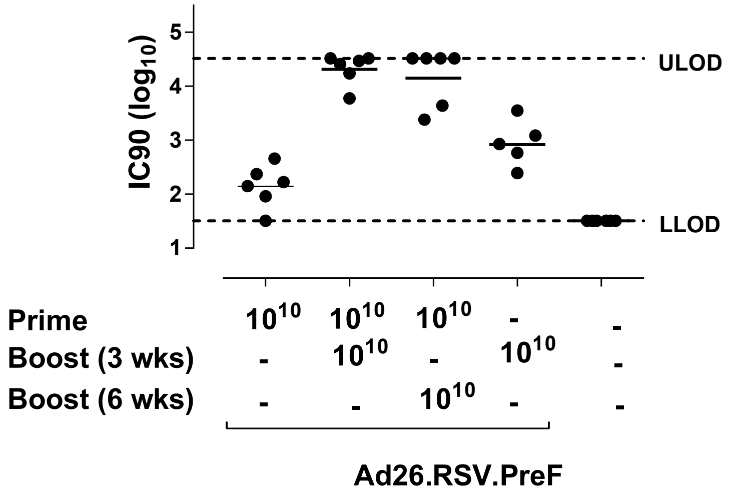

Supplementary Figure 9: Anti-Ad26 neutralizing antibody titers are strongly boosted by a second homologous immunization. Pups were subcutaneously immunized with 10^{10} vp Ad26.RSV.preF in the immunization regimens displayed (n=6 per group). Control groups received no immunization (indicated with -). Ad26 neutralizing antibody titers were determined in serum isolated 25 weeks post prime immunization. Titers are given as the $\log_{10}$ value of the IC90. Horizontal bars depict the mean of the response within a group. The Lower Limit of Detection (LLOD) and Upper Limit of Detection (ULOD) of the assay are indicated with dashed lines.

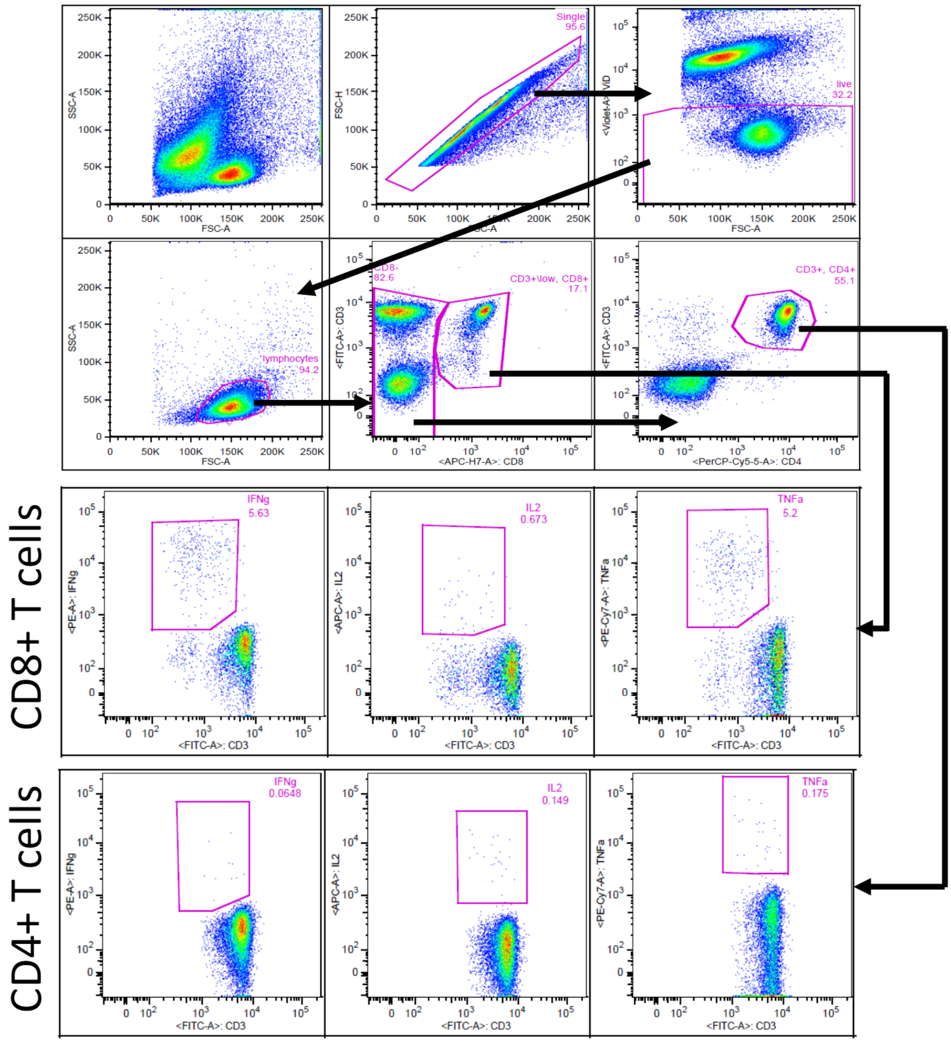

Supplementary Figure 10: Gating strategy used to identify RSV-F specific T cells in splenocytes by intracellular cytokine staining and flow cytometry. Exemplified strategy demonstrating identification of lymphocytes based on forward and side scatter (FSC and SSC) characteristics after exclusion of doublets based on their FSC-area/FSC-height characteristics, and exclusion of dead cells based on positivity for the Live/Dead cell discrimination fluorochrome (amine reactive dye, Violet-A-ViD). Cells were gated based on expression of CD3, CD4 or CD8. Gates for the expression of IFNγ, TNFα, or IL-2 in gated CD8+ or CD4+ cells were set based on fluorescence-minus-one controls (FMO-controls). This gating strategy was used to determine the % of CD4+ or CD8+ cells positive for the different cytokines, that are displayed in Figure 4C, and Supplementary Figures 2 and 6.
